# VALIDITY AND RELIABILITY OF A NEW TRIAGE SYSTEM FOR PEDIATRIC EMERGENCY CARE: CLARIPED

**DOI:** 10.1590/1984-0462/;2018;36;4;00017

**Published:** 2018

**Authors:** Maria Clara de Magalhães-Barbosa, Arnaldo Prata-Barbosa, Carlos Eduardo Raymundo, Antonio José Ledo Alves da Cunha, Claudia de Souza Lopes

**Affiliations:** a Instituto D’Or de Pesquisa e Ensino, Rio de Janeiro, RJ, Brasil.; b Universidade Federal do Rio de Janeiro, Rio de Janeiro, RJ, Brasil.

**Keywords:** Emergency medical services, Pediatrics, Triage, Risk assessment, Reproducibility of results, Serviços médicos de emergência, Pediatria, Triagem, Medição de risco, Reprodutibilidade dos resultados

## Abstract

**Objective:**

To assess the validity and reliability of a triage system for pediatric emergency care (CLARIPED) developed in Brazil.

**Methods:**

Validity phase: prospective observational study with children aged 0 to 15 years who consecutively visited the pediatric emergency department (ED) of a tertiary hospital from July 2 to 18, 2013. We evaluated the association of urgency levels with clinical outcomes (resource utilization, ED admission rate, hospitalization rate, and ED length of stay); and compared the CLARIPED performance to a reference standard. Inter-rater reliability phase: a convenience sample of patients who visited the pediatric ED between April and July 2013 was consecutively and independently double triaged by two nurses, and the quadratic weighted kappa was estimated.

**Results:**

In the validity phase, the distribution of urgency levels in 1,416 visits was the following: 0.0% red (emergency); 5.9% orange (high urgency); 40.5% yellow (urgency); 50.6% green (low urgency); and 3.0% blue (no urgency). The percentage of patients who used two or more resources decreased from the orange level to the yellow, green, and blue levels (81%, 49%, 22%, and 2%, respectively, *p*<0.0001), as did the ED admission rate, ED length of stay, and hospitalization rate. The sensitivity to identify patients with high urgency level was 0.89 (confidence interval of 95% [95%CI] 0.78-0.95), and the undertriage rate was 7.4%. The inter-rater reliability in 191patients classified by two nurses was substantial (kw^2^=0.75; 95%CI 0.74-0.79).

**Conclusions:**

The CLARIPED system showed good validity and substantial reliability for triage in a pediatric emergency department.

## RESUMO

**Objetivo:**

Avaliar a validade e a confiabilidade de um sistema de classificação de risco para emergências pediátricas (CLARIPED) desenvolvido no Brasil.

**Métodos:**

Fase de validade: estudo observacional prospectivo em crianças de 0 a 15anos atendidas consecutivamente no serviço de emergência pediátrica (SEP) de um hospital terciário entre 2 e 18de julho de 2013. Avaliou-se a associação dos níveis de urgência com desfechos clínicos (utilização de recursos, taxa de admissão no SEP, taxa de hospitalização e tempo de permanência no SEP); e comparou-se o desempenho do CLARIPED com um padrão de referência. Fase de confiabilidade interobservadores: uma amostra de conveniência de pacientes atendidos entre abril e julho de 2013 foi submetida a duas classificações de risco consecutivas e independentes, e estimou-se o Kappa ponderado quadrático.

**Resultados:**

Em 1.416 atendimentos realizados na fase de validade, a distribuição de níveis de urgência foi: 0,0% vermelho/emergência; 5,9% laranja/muito urgente; 40,5% amarelo/urgente; 50,6% verde/pouco urgente; e 3,0% azul/sem urgência. Ospercentuais de pacientes que usaram dois ou mais recursos diminuíram do nível laranja para o amarelo, verde e azul (81, 49, 22 e 2%, respectivamente, *p*<0,0001), assim como a taxa de admissão no SEP, o tempo de permanência no SEP e a taxa de admissão hospitalar. Asensibilidade para discriminar pacientes muito urgentes foi de 0,89 (intervalo de confiança de 95%- IC95% 0,78-0,95), e a taxa de subtriagem foi de 7,4%. Aconfiabilidade interobservadores em 191 pacientes classificados por duas enfermeiras foi substancial (kw^2^=0,75; IC95% 0,74-0,79).

**Conclusões:**

O sistema CLARIPED mostrou boa validade e substancial confiabilidade para classificação de risco em um serviço de emergência pediátrica.

## INTRODUCTION

Triage in the pediatric emergency department (ED) is a challenge. Limited ability to communicate, subclinical presentations in young children, variations in normal vital signs (VS) according to age group, among other factors, make pediatric triage a complex and difficult task.^1^

The triage systems most commonly used worldwide for pediatric emergency care are the Manchester Triage System (MTS), the Canadian Pediatric Triage and Acuity Scale (PedCTAS), the Emergency Severity Index (ESI), and the Australasian Triage Scale (ATS).^2^ ^,^ ^3^ These instruments were originally designed for adults, and later adapted for children, who represent 20 to 40% of the population treated in emergency departments.^4^

The validity and reliability of these triage systems for children have been assessed predominantly in the countries they were created or in developed countries with similar cultures.^5^ ^,^ ^6^ ^,^ ^7^ ^,^ ^8^ ^,^ ^9^ ^,^ ^10^ ^,^ ^11^ ^,^ ^12^ ^,^ ^13^ ^,^ ^14^ ^,^ ^15^ ^,^ ^16^ ^,^ ^17^ ^,^ ^18^ ^,^ ^19^ ^,^ ^20^ ^,^ ^21^ ^,^ ^22^ These instruments are very extensive or complex, and their performance in countries with distinct sociodemographic and/or cultural characteristics has been lower than in their original countries.^23^ ^,^ ^24^ ^,^ ^25^ ^,^ ^26^ Differences in human and technological resources, professional qualification, and health policies can interfere in their performance. Simpler algorithms, provided they are valid and reliable, could be more appropriate for countries like Brazil.

The South African Triage Scale(SATS) is a simple and objective tool;^27^ however, it has only four urgency levels (ULs) and three age groups for VS evaluation. The tool recommended by the World Health Organization (WHO) for less developed countries, the Emergency Triage Assessment and Treatment (ETAT), prioritizes the identification of patients with a high urgency level.^28^ This characteristic does not reflect 70 to 90% of the population who crowds the public and private Brazilian pediatric EDs and have intermediate urgency levels.

To meet these demands, a team of pediatric ED experts in Brazil has developed a five-level triage system for pediatric emergency care (CLARIPED), which is simple and objective, easy to use and train, and stratified into five age groups.^29^ Thepurpose of this study was to assess the validity and reliability of this instrument.

## METHOD

This is a prospective observational study conducted in the pediatric ED of a private tertiary hospital in the city of Rio de Janeiro (Rio de Janeiro, Brazil), with 3,000 visits per month, daily staff of 3 to 4 doctors, 2 nurses, and 2 nurse technicians, in addition to pediatric residents.

To assess the validity of CLARIPED, all consecutive patients who visited the ED and underwent triage from July 2 to 18, 2013 were included. We excluded patients who did not undergo triage. Data were collected every 24 hours from the medical records of the previous day. Demographic and clinical variables from triage and during ED stay, such as diagnostic/therapeutic resources, ED length of stay, and destination were collected to digital forms. Data were reviewed for consistency.

To evaluate inter-rater reliability, we prospectively selected a convenience sample during daytime shifts (8 to 17h) between April and July 2013. Immediately after the conventional triage performed by the regular triage nurse, consecutive patients and their guardians were invited to voluntarily participate in the study. If they agreed and the guardian signed the Informed Consent Form, they were taken to another room, where a research nurse blind to the first classification, performed a new complete triage procedure. The research nurses belonged to the triage team, had the same level of training, and voluntarily participated in the study, in their extra work hours. In this phase, for ethical reasons, we excluded patients who needed immediate treatment, due to the impossibility of subjecting them to two consecutive triage procedures.

CLARIPED was applied as previously described.^29^ The first step was the assessment of four vital signs to calculate the pediatric vital signs score (VIPE score), from 0 to 12, classified in five ULs: 0=blue (no urgency); 1 or 2=green (low urgency); 3to 5=yellow (urgency); 6 to 9=orange (high urgency); and 10 to 12=red (emergency). The second step was the evaluation for the presence of clinical discriminators consisting of signs, symptoms and/or complaints, also distributed into 5 ULs. Ifan identified discriminator corresponded to a higher UL than the one determined by the VIPE score, the final classification would be the one with greatest UL.^29^

Due to the lack of a gold standard for triage in pediatric ED, we used two methods to evaluate validity:

1. the association between the UL designated by CLARIPED and four clinical outcomes (diagnostic/therapeutic resource utilization, admission rate at the ED observation room, ED length of stay, and hospitalization), which were considered proxies of urgency, similarly to other studies;^9^ ^,^ ^10^ ^,^ ^11^ ^,^ ^12^ ^,^ ^14^ ^,^ ^15^ and
2. comparison between the CLARIPED classification and the one determined by a reference standard.

The first method was based on the following hypothesis: if CLARIPED adequately identifies the five ULs, a decreasing gradient in the frequency of outcomes should occur, from the highest to the lowest UL. The outcome “resource utilization” included diagnostic tests, therapeutic procedures, and specialty consultations, according to a previously standardized and adapted table from Gilboy etal*.* ^*30*^ This variable was dichotomized (<2 resources and ≥2 resources), similarly to other studies.^12^ ^,^ ^14^ ^,^ ^15^ ^,^ ^19^ The admission rate to the ED observation room comprised only children who, after occupying an ED bed, were discharged home. ED length of stay was calculated from the beginning of physician assessment until the patient left the ED. Patients who progressed to hospitalization, even those transferred to other institutions, were included in the hospitalization rate.

In the second method, the reference standard was based on a matrix developed by experts to study the MTS in the pediatric population^19^ and adapted for the present study. This matrix used data extracted from medical records (significant vital signs changes, life-threatening clinical conditions, laboratory and imaging tests, therapeutic approach, and patient destination), alone or in various possible combinations, to retrospectively identify the appropriate urgency level and compare it with the one previously assigned by the triage system.

For the validity, we estimated a sample of 1,385 ED visits, based on data from the literature regarding the ED length of stay, which was the outcome that demanded the largest sample. Assuming an alpha error=0.05 and beta error=0.80, we used the difference of 71 minutes between level 2 (high urgency) and level 3 (urgency), reported in a study on the ESI-4 (309minutes; 95%CI 257-361, SD=225.5 versus 238minutes; 95%CI223-251, SD=112.8, respectively).^15^

For the inter-rater reliability, the sample calculation was based on a pilot study including 61 visits, which generated a quadratic weighted Kappa (kw^2^) of 0.57 (95%CI ­0.51-0.68). To reduce the confidence interval range to 0.10, we made simulations with increasing samples sizes, and the same distribution of agreements and disagreements between ULs observed in the pilot study, resulting in an estimated sample of 183 visits.

Associations between ULs and outcomes were assessed using the chi-square test or Fisher’s exact test for categorical variables and the Mann-Whitney test or Kruskal-Wallis test for continuous variables. We used logistic regressions to estimate odds ratios (OR) resulting from the association of ULs (independent variables) with hospitalization and use of resources (dependent variables), after adjustments for potential confounding factors (age, service time and day of the week). Overtriageand undertriage rates, sensitivity, and specificity in diagnosing high urgency cases were calculated by comparing CLARIPED with the reference standard. Stratification by age group and diagnostic categories were performed on an exploratory basis.

For inter-rater reliability, we chose kw^2^ because this estimate takes into account the degree of disagreement between categories, in addition to being the most widely used in other studies.

The analysis considered a significance level of 0.05 and 95%CI. We used the statistical softwares Stata 12.0 (StataCorp, College Station, Texas, United States) and R 2.15.3 (R Foundation, Vienna, Austria). The Committee for Ethics in Research (CER) of the institution approved this project.

## RESULTS

**Figure 1:**
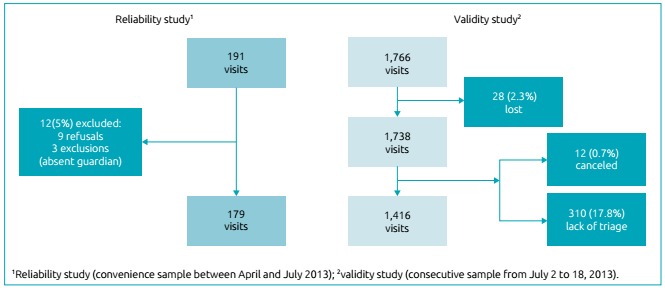
Patient selection algorithm for the validity and reliability studies.

The validity phase included 1,416 consecutive visits (80.2% of those eligible) and excluded 28 cases whose medical records were lost (1.6%), 12 cases who left the ED before triage (0.7%), and 310 cases who did not undergo triage (17.6%). In the reliability phase, 179 patients agreed to participate in the study (93.7% of invitees), 9 refused, and 3 were excluded due to the absence of a legal guardian (Figure 1).

The validity sample had the following distribution: red 0.0%; orange 5.9%; yellow 40.5%; green 50.6%; and blue 3.0% (Table 1). Resource utilization was evaluated in 1,415 visits, while admission to the ED observation room and hospitalization were evaluated in 1,413 visits and ED length of stay in 1,090 visits (Table 2). The 326 cases with missing data for this last outcome showed an UL distribution similar to the 1,416 included cases (*p*=0.2181), suggesting missing at random loss. The frequencies of all outcomes decreased as the ULs lowered: ≥2 resources (orange 80.9%, yellow 48.5%, green 21.8%, and blue 2.4%; *p*<0.0001), admission to the ED observation room (20.2%, 4.7%, 0.4%, and 0.0%; *p*=0.0005), hospitalization (19.0%, 2.6%, 0.0%, and 0.0%; *p*=0.0005), and ED length of stay; (209, 106, 47, and 27 minutes; *p*<0.0001) (Table 2).

**Table 1:** Baseline characteristics of the general population and validity and reliability samples, and triage of validity and reliability samples.

Characteristics	Total population assisted between April and July 2013	Participants of the validity assessment*	Non-participants of the validity assessment	Participants of the reliability assessment*
Total	13,453	1,416	322	179
Male	6,917 (51.4)	719 (50.8)	199 (61.8)	94 (52.5)
Age group (years)				
< 1	† 6,336 (47.1)	145 (10.2)	41 (12.7)	17 (9.5)
1-4		723 (51.1)	148 (46.0)	84 (46.9)
5-11	5,304 (39.4)	459 (32.4)	95 (29.5)	59 (33.0)
12-15	1674 (12.4)	89 (6.3)	38 (11.8)	19 (10.6)
NA	139 (1.0)	0	0	0
Visit period				
7-13h	4,408 (32.8)	491 (34.7)	57 (17.7)	159 (88.8)
13-19h	4670 (34.7)	532 (37.6)	79 (25.5)	20 (11.2)
19-1h	3,391 (25.2)	314 (22.2)	145 (45.0)	0
1-7h	746 (5.5)	79 (5.6)	41 (12.7)	0
NA	238 (1.8)	0	0	0
Days of the week				
Monday	2,207 (16.4)	203 (14.3)	35 (10.9)	21 (11.7)
Tuesday	1,919 (14.3)	239 (16.9)	54 (16.8)	10 (5.6)
Wednesday	1,870 (13.9)	242 (17.1)	51 (15.8)	82 (45.8)
Thursday	1,717 (12.8)	232 (16.4)	475 (14.6)	18 (10.1)
Friday	1,689 (12.6)	146 (10.3)	22 (7.1)	48 (26.8)
Saturday	1,993 (14.8)	170 (12.0)	63 (19.6)	0
Sunday	2,058 (15.3)	184 (13.0)	49 (15.2)	0
Main final diagnoses				
Upper respiratory disease		466 (32.9)	82 (25.5)	61 (34.1)
Lower respiratory disease		179 (12.6)	41 (12.7)	38 (21.2)
Digestive		179 (12.6)	23 (7.1)	16 (8.9)
Ear diseases		134 (9.5)	37 (11.5)	19 (10.6)
External causes		148 (10.5)	44 (13.7)	2 (1.1)
General and unspecific		113 (8.0)	26 (8.1)	8 (4.5)
Destination				
Discharge after prescription		776 (54.8)	164 (50.9)	115 (64.2)
Discharge after medication		559 (39.5)	99 (30.7)	58 (32.4)
Discharge after observation		47 (3.3)	17 (5.3)	6 (3.4)
Room hospitalization	‡ 279 (2.1)	Ⱡ 17 (1.2)	23 (7.1)	0
PICU hospitalization		Ⱡ 4 (0.3)	2 (0.6)	0
Transfer	39 (0.3)	Ⱡ 10 (0.7)	3 (0.9)	0
Evasion rate before consultation	129 (1.0)		-	
Discharge against medical adviceafter consultation	189 (1.2)		-	
NA	89 (0.7)	3 (0.2)	14 (4.3)	0
Urgency level				1st/2nd¤
Red		0		0/0
Orange		84 (5.9)		13 (7.3)/13 (7.3)
Yellow		573 (40.5)		70 (39.1)/75 (41.9)
Green		717 (50.6)		75 (41.9)/62 (34.6)
Blue		42 (3.0)		21 (11.7)/29 (16.2)

*Consecutive sample; **convenience sample; NA: none of the alternatives; PICU: pediatric intensive care unit; †grouping the visits of all children <5 years; ‡grouping all room and PICU hospitalizations=2.1%; Ⱡadding up room hospitalizations (1.2%), PICU hospitalizations (0.3%), and transfers (0.7%), the total hospitalization rate=2.2%; 1st and 2nd triages of the reliability assessment.

**Table 2:** Distribution of progressive outcomes for different urgency categories: validity study (n=1,416).

Urgency level	Therapeutic approach and destination				
	Total n (%)	≥2 resources^1^ n (%)	Admission to the observation room^2^ n (%)	Hospitalization^3^ n (%)	Average length of stay^4^ min (95%CI)
Red	0 (0.0)	-	-	-	-
Orange	84 (5.9)	68 (81.0)	17 (20.2)	16 (19.0)	209 (160-258)
Yellow	573 (40.5)	278 (48.5)	27 (4.7)	15 (2.6)	106 (93-119)
Green	717 (50.6)	125 (21.8)	3 (0.4)	0	47 (43-51)
Blue	42 (3.0)	1 (2.4)	0 (0.0)	0	27 (19-35)
Total	1,416 (100.0)	472 (33.4)	47 (3.3)	31 (2.2)	
p-value		*<0.0001	**0.0005	**0.0005	***<0.0001

*Chi-square test; **Fisher’s exact test; ***Kruskal-Wallis test; ^1^total of 1,415 visits evaluated; ^2^total of 1,413 visits evaluated; ^3^total of 1,413visits evaluated; ^4^total of 1,090 visits evaluated; 95%CI: confidence interval of 95%.

Orange level patients had a higher chance of using ≥2resources (OR=4.67; 95%CI 2.61-8.34, adjusted for age and service time) and greater chance of hospitalization (OR=10.77; 95%CI 4.85-23.91, adjusted for age), when compared to yellow level patients.

The comparison between CLARIPED and the reference standard showed absolute agreement in 33.5% of cases, overtriage in 59.1%, and undertriage in 7.4%. Most of the disagreements represented assignments one category above the correct classification (49.4%), mainly in the green and blue levels, or below it (7.3%), mainly in the yellow level (Table 3). There were no differences between age groups in overtriage (*p*=0.20 to 0.98) and undertriage (*p*=0.13 to 0.52) when compared to general rates. Overtriage rates were lower for lower respiratory diseases (29.6%; *p*<0.01), and higher for upper respiratory diseases (67.1%; *p*=0.002) and ear diseases (76.1%; *p*=0.0002). No diagnostic category showed an undertriage rate different from the general one of 7.4%.

**Table 3:** Urgency level assigned by the reference standard versus triage system for pediatric emergency care (CLARIPED): validity study (n=1,416).

CLARIPED	Reference standard					
	Red	Orange	Yellow	Green	Blue	Total
Red	0	0	0	0	0	0
Orange	0	55	13	12	4	84
Yellow	0	6	175	271	121	573
Green	0	1	86	214	416	717
Blue	0	0	0	11	31	42
Total	0	62	274	508	572	1,416
Absolute agreement Undertriage of 1 category Overtriage of 1 category			Undertriage >1 category Overtriage >1 category			
Absolute agreement = 33.5% (475/1,416)						
Total overtriage = 59.1% (837/1,416)			Total undertriage = 7.3% (104/1,416)			
Overtriage (orange) = 0.0%			Undertriage (orange) = 11.3% (7/62)			
Overtriage (yellow) = 4.7% (13/274)			Undertriage (yellow) = 31.4% (86/274)			
Overtriage (green) = 55.7% (283/508)			Undertriage (green) = 2.2% (11/508)			
Overtriage (blue) = 94.5% (541/1,416)			Undertriage (blue) = 0			

CLARIPED sensitivity and specificity in identifying high urgency levels were 0.89 (95%CI 0.78-0.95) and 0.98 (95%CI 0.97-0.99), respectively. The stratification of these estimates by diagnostic categories and age group was impaired due to the small number of cases in subgroups (Table 4).

**Table 4:** CLARIPED sensitivity and specificity in identifying high urgency in the general population, age range subgroups, and main diagnostic categories.

Subgroup	No. of patients	% of high urgency*		Sensitivity^†^	Specificity^†^
		CLARIPED	Reference		
Total	1,416	84 (5.9%)	62 (4.4%)	0.89 (0.78-0.95)	0.98 (0.97-0.99)
Age group (years)					
<1	145	9 (6.2%)	9 (6.2%)	0.89 (0.52-1.00)	0.99 (0.96-1.00)
1-4	723	31 (4.3%)	25 (3.5%)	0.84 (0.64-0.95)	0.99 (0.97-0.99)
5-11	459	34 (7.4%)	22 (4.8%)	0.91 (0.71-0.99)	0.97 (0.95-0.98)
12-15	89	10 (11.2%)	6 (6.7%)	1.00 (0.42-1.00)	0.95 (0.88-0.99)
Final diagnosis					
Upper respiratory	466	5 (1.1%)	4 (1.0%)	0.75 (0.19-0.99)	1.00 (0.98-1.00)
Lower respiratory	179	37 (20.7%)	31 (17.3%)	1.00 (0.84-1.00)	0.96 (0.92-0.99)
Digestive	179	17 (9.5%)	14 (7.8%)	0.79 (0.49-0.95)	0.96 (0.92-0.99)
Ear	134				
External causes	148	11 (7.4%)	2 (1.4%)	1.00 (0.09-1.00)	0.94 (0.89-0.97)
General and unspecific	113	4 (3.5%)	4 (3.5%)	0.50 (0.07-0.93)	0.98 (0.94-1.00)

*high urgency: red and orange categories; sensitivity^†^: cases of high urgency (red and orange) designated by CLARIPED/cases of high urgency determined by the reference standard; specificity^†^: cases of low urgency (yellow, green, and blue) designated by CLARIPED/cases of low urgency determined by the reference standard.

In the reliability phase, 15 nurses with the same training level on CLARIPED participated in pairs in the double triage: 13 nurses in the first and two nurses in the second triage. Themedian age of the nurses was 28 years old (interquartile range [IQR]: 26.0-29.5). Four nurses had over five years of pediatric ED experience, including the two research nurses, while 11 nurses had less than five years of experience. The UL distribution in the first triage was orange 7.3%, yellow 39.1%, green 41.9%, and blue 11.7%; and in the second triage was orange 7.3%, yellow 41.9%, green 34.6%, and blue 16.2% (Table 1). The absolute agreement was 68.7%, and the kw^2^ was 0.75 (95%CI 0.73-0.79) (Table5).

**Table 5:** Urgency level determined by the research nurse versus triage nurse - reliability study (n=179).

Triage nurse	Research nurse					
	Red	Orange	Yellow	Green	Blue	Total
Red	0	0	0	0	0	0
Orange	0	10	3	0	0	13
Yellow	0	3	56	11	0	70
Green	0	0	15	44	16	75
Blue	0	0	1	7	13	21
Total	0	13	75	62	29	179
Absolute agreement = 68.7%; kw^2^=0.75 (95%CI 0.73-0.79)						

kw^2^: Quadratic weighted kappa; 95%CI: confidence interval of 95%.

## DISCUSSION

In addition to having good validity and reliability, an ideal triage system must be feasible and effective. Ensuring team adherence and procedure expedition is essential. CLARIPED showed good validity, demonstrated by a strong association between ULs and clinical outcomes, in addition to substantial inter-rater reliability. The measures of association with outcomes were comparable to those observed in similar studies with other triage systems. The chance of hospitalization in the orange level was almost 11 times higher than in the yellow and greater than the estimate of a multicenter study on PedCTAS (OR=4.93; 95%CI 2.95-8.25).^10^ However, in the present study, the hospitalization rate (2.2%) was lower than those reported in other studies (5-10%),^9^ ^,^ ^10^ ^,^ ^12^ ^,^ ^15^ ^,^ ^26^ ^,^ ^27^ which could be the result of differences in populations or institutional policies.

Given the low hospitalization rate, the resource utilization was a more appropriate outcome for the population under study. The frequency of patients who used ≥2 resources decreased from highest to lowest UL (81.0; 48.5; 21.8; and 2.4%; *p*<0.0001). Considering that there were no patients classified as red, these results were similar to those reported with the ESI-4 (100, 70, 45, 17, and 4%)^14^ and are more discriminant than those found with the MTS (41.7, 25.4, 30.2, 16.6, and 3.7%).^19^ The orange level showed an almost 5 times higher chance of using ≥ 2 resources when compared to the yellow level, while the green level showed a 5 times lower chance. This association was also very close to that reported with the PedCTAS, when comparing high urgency level (OR=4.67; 95%CI 2.61-8.34) and low urgency level (OR=0.21; 95%CI 0.17-0.28) to the urgency level cases as reference.^10^

The pediatric ED length of stay was calculated from the beginning of the assessment by a doctor, and not on arrival at the ED, as in most studies. The purpose was to avoid distortion in the association between this outcome and the UL since the triage process determines that the lower the urgency level assigned to the patient, the higher the waiting time to be seen by a doctor. The distribution of length of stay showed a decreasing gradient from the highest to the lowest UL, corroborating the good validity of the instrument (209, 106, 47, and 27 minutes; *p*<0.0001). Disregarding the level 1 (red), this gradient was also more discriminant than those identified in two studies on the PedCTAS (191, 250, 191, 96, and 66 minutes; *p*<0.0001, and 309, 238, 186, and 160 minutes),^9^ ^,^ ^10^ and two studies on the ESI-4 (334, 221, 207, 151, and 132minutes; *p*<0.001, and 156, 236, 259, 117, and 99minutes; *p*<0.0001).^12^ ^,^ ^14^ However, the difference in the definition of this outcome in these studies might have contributed to the less consistent results.

Despite the difference in sample size between this study (n=1,416) and two studies on the MTS (n=13,554 and 11,260),^20^ ^,^ ^22^ the use of a similar reference standard by the three studies allows some comparisons between the performance of the tools. CLARIPED showed absolute agreement similar to MTS (33.5 versus 34.0%); higher overtriage rate (59.1% versus 54.0%), higher sensitivity (89.0 versus 63.0%), and specificity (98.0 versus 79.0%); and lower undertriage rate (7.4% versus 12.0%).^20^ After modifications in some MTS pediatric discriminators, specificity increased (87%), overtriage decreased (47%), sensitivity did not change (64%), and undertriage presented a slight increase (15%).^22^ Nonetheless, it is important to question whether the reference standard provides the appropriate urgency level in all cases. For example, an infant who arrives at the pediatric ED weeping, with irritability, and intense pain would be properly classified as orange or yellow by MTS and CLARIPED. If the final diagnosis is acute otitis media, which is a very common entity in pediatrics, the patient will be medicated with an analgesic and discharged with a prescription, being considered green by the reference standard. Actually, the CLARIPED overtriage rate was particularly high for ear diseases. In the same way, a patient with an extensive cut-contusion wound, requiring sutures, would be classified as yellow by CLARIPED and MTS, and green by the reference standard. These and other similar cases could justify the low absolute agreement, as well as the high overtriage rate of both tools compared to the reference standard. In fact, the reference standard was not validated.

This study estimated the reliability by including only patients treated in real time, instead of hypothetical scenarios, which were commonly used in several studies.^5^ ^,^ ^12^ ^,^ ^16^ ^,^ ^17^ ^,^ ^21^ ^,^ ^27^ Clinical scenarios do not replicate the difficulties of the actual triage process, being subjected to biases. The present study exhibited substantial inter-rater reliability (kw^2^=0.75; 95%CI 0.74-0.79). Thisresult is better than those obtained in the first studies on other instruments with actual patients: MTS (kw^2^=0.65; 95%CI 0.56-0.72),^20^ PedCTAS (kw^2^=0.61; 95%CI 0.42-0.80),^8^ and ESI-4 (kw^2^=0.57; 95%CI 0.52-0.62).^14^ More recent studies showed better reliability with PedCTAS,^10^ (kw^2^=0.74; 95%CI 0.71-0.76) and ESI-4^15^ ^,^ ^26^ (k­linear=0.92; p<0.001 and k not specified=0.82; 95%CI 0.67-0.84). The improvement in reliability over time probably reflects the refinement of these tools and a progressive better qualification of the teams. In this regard, the reliability exhibited by the first version of CLARIPED is promising.

This study has some limitations. It was carried out in a single center, and the researchers could have over-motivated the health team, resulting in an overestimation of the validity and reliability of CLARIPED. However, the easy assimilation and implementation of the tool suggest that it could be appropriate for many similar environments, including non-hospital emergency departments.

Another limitation was that participants of the validity phase represented 80.2% of eligible patients and the hospitalization rate was higher among non-participants (8.6%versus2.2%)(Table1). The most plausible reason for this difference is that the pediatric ED of the present study receives patients referred to hospitalization by its assistant pediatricians. These children are sent directly to the ED observation room to start treatment, without undergoing triage; however, the characteristics of the study participants did not differ from the total pediatric ED population (Table1).

An additional limitation is the lack of patients classified as red in the period studied; however, this fact does not invalidate the results found in the other four urgency levels, which constitute about 99% of emergency pediatric care. Two validity studies on PedCTAS did not include patients requiring immediate care either.^6^ ^,^ ^10^ These patients are very rare in most pediatric EDs around the world,^7^ ^,^ ^9^ ^,^ ^11^ ^,^ ^23^ ^,^ ^25^ ^,^ ^26^ and, in daily practice, they do not undergo triage, being directly led to the reanimation room, and classified retrospectively. On the other hand, one of the main challenges of triage system is discriminating intermediate UL patients, such as levels 3 (urgency) and 4 (low urgency), which comprise the vast majority of patients who crowd the pediatric EDs. Level 3 patients are those with the potential to have their condition worsen if they wait a long time for medical care, but who might not be easily identified without an objective assessment. Surgical abdominal pain (appendicitis or intussusception), cases with the risk of severe dehydration (profuse diarrhea or incoercible vomiting), or acute bacterial infection (high fever in small children) are some examples of level 3 (urgent) patients.

Lastly, the present study used clinical outcomes as proxies of urgency to determine the convergent construct validity. However, the goal of triage systems is not to predict clinical outcomes, which are good markers of complexity and severity of diseases, but do not always reflect the level of urgency in all situations, in addition to being influenced by the quality of treatment and institutional policies. For instance, a patient with seizures (red) or having an asthma crisis (orange) can be discharged from the ED observation room a few hours after treatment, without needing hospitalization. On the other hand, a patient with a serious chronic disease can come to the ED with a low urgency complication (green) and need hospitalization due to the underlying disease.

In conclusion, this is the first study on the validity and reliability of a pediatric triage system in Brazil. CLARIPED proved to be a valid and reliable instrument in the center where it was developed. A multicenter study is necessary to corroborate these preliminary findings, indicate the adjustments needed for different health contexts, and assess the external validity of the instrument.
